# Cell Surface Localization of α3β4 Nicotinic Acetylcholine Receptors Is Regulated by N-Cadherin Homotypic Binding and Actomyosin Contractility

**DOI:** 10.1371/journal.pone.0062435

**Published:** 2013-04-23

**Authors:** Juan L. Brusés

**Affiliations:** 1 Department of Anatomy and Cell Biology, University of Kansas School of Medicine, Kansas City, Kansas, United States of America; 2 Department of Psychiatry and Behavioral Sciences, University of Kansas School of Medicine, Kansas City, Kansas, United States of America; Virginia Tech Carilion Research Institute, United States of America

## Abstract

Neuronal nicotinic acetylcholine receptors (nAChRs) are widely expressed throughout the central and peripheral nervous system and are localized at synaptic and extrasynaptic sites of the cell membrane. However, the mechanisms regulating the localization of nicotinic receptors in distinct domains of the cell membrane are not well understood. N-cadherin is a cell adhesion molecule that mediates homotypic binding between apposed cell membranes and regulates the actin cytoskeleton through protein interactions with the cytoplasmic domain. At synaptic contacts, N-cadherin is commonly localized adjacent to the active zone and the postsynaptic density, suggesting that N-cadherin contributes to the assembly of the synaptic complex. To examine whether N-cadherin homotypic binding regulates the cell surface localization of nicotinic receptors, this study used heterologous expression of N-cadherin and α3β4 nAChR subunits C-terminally fused to a myc-tag epitope in Chinese hamster ovary cells. Expression levels of α3β4 nAChRs at cell-cell contacts and at contact-free cell membrane were analyzed by confocal microscopy. α3β4 nAChRs were found distributed over the entire surface of contacting cells lacking N-cadherin. In contrast, N-cadherin-mediated cell-cell contacts were devoid of α3β4 nAChRs. Cell-cell contacts mediated by N-cadherin-deleted proteins lacking the β-catenin binding region or the entire cytoplasmic domain showed control levels of α3β4 nAChRs expression. Inhibition of actin polymerization with latrunculin A and cytochalasin D did not affect α3β4 nAChRs localization within N-cadherin-mediated cell-cell contacts. However, treatment with the Rho associated kinase inhibitor Y27632 resulted in a significant increase in α3β4 nAChR levels within N-cadherin-mediated cell-cell contacts. Analysis of α3β4 nAChRs localization in polarized Caco-2 cells showed specific expression on the apical cell membrane and colocalization with apical F-actin and the actin nucleator Arp3. These results indicate that actomyosin contractility downstream of N-cadherin homotypic binding regulates the cell surface localization of α3β4 nAChRs presumably through interactions with a particular pool of F-actin.

## Introduction

Neuronal nicotinic acetylcholine receptors (nAChRs) are ligand-gated ion channels comprised of five homologous subunits arranged around a central pore. They belong to the superfamily of cys-loop receptors due to the presence of an extracellular pair of disulphide-bonded cysteines separated by a stretch of 13 amino acids [1]. Eleven nAChR subunits have been identified in mammalian neurons (α2 to α10 and β2 to β4) [2], [3]. All nAChR subunits have a similar structure with an N-terminal extracellular domain of ∼210 amino acid residues containing the acetylcholine binding site, four transmembrane domains (TM1–TM4), a large intracellular loop of ∼150 amino acids between TM3 and TM4, and a short extracellular C-terminal tail [4]. Neuronal nAChRs are homo (α subunits) or hetero (α and βsubunits) pentamers widely expressed in the central and peripheral nervous system. Neuronal nAChRs with different subunit compositions localize to distinct domains of the cell surface including pre and postsynaptic membranes, and extrasynaptic sites [5]. Various mechanisms have been identified regulating the localization of nAChRs at distinct domains of the cell membrane including, targeting of the receptors to dendrites and axons [6], incorporation into cholesterol-rich membrane microdomains [7], and association of receptor subunits with scaffolding proteins and the actin cytoskeleton [8]–[10]. Heteromeric neuronal nAChRs comprise of α3 and β4 subunits are abundant in the peripheral nervous system and drive excitatory cholinergic neurotransmission in autonomic ganglia [4], [8], [11]. Neuronal nAChRs containing α3 subunits are localized at the postsynaptic density apposed to presynaptic active zones similarly to glutamate receptors in excitatory synapses of the central nervous system [12]. However, the mechanisms regulating the localization of α3β4 nAChRs on the cell membrane and their assembly into receptor clusters are not entirely understood.

Cell adhesion molecules expressed at synaptic contacts play important roles in the induction of synapse formation, in the stabilization of the contact between synaptic membranes, and in synaptic plasticity [13]–[15]. N-cadherin is a transmembrane cell adhesion molecule that mediates homotypic binding across the extracellular space of contacting cell membranes and regulates the actin cytoskeleton by interacting with p120-catenin and β-catenin, and by regulating small Rho GTPases [16]–[20]. N-cadherin is abundantly expressed at glutamatergic and cholinergic synaptic contacts from the early stages of synapse formation [21]–[24]. Within the synaptic complex, N-cadherin commonly forms aggregates or puncta that are localized adjacent to the active zone and the postsynaptic density [21], [23]–[25], suggesting that in addition to providing adhesive support to the synaptic contact it contributes to the assembly of synaptic components. Indeed, N-cadherin regulates glutamate receptor trafficking and stability on the cell surface [26], [27] and recruits synaptogenic molecules and postsynaptic scaffolding proteins to synaptic contacts [28]–[31]. In addition, N-cadherin may affect the localization and distribution of components of the synaptic complex by regulating the actin cytoskeleton via Rho GTPases.

This study was aimed at determining whether N-cadherin homotypic binding and the actin cytoskeleton regulate the cell surface localization of neuronal nAChRs comprised of the α3 and β4 subunits (α3β4 nAChRs). Heterologous expression of N-cadherin and α3β4 nAChRs in non-neuronal cell lines was used to examine the cell surface localization of α3β4 nAChRs with respect to N-cadherin-mediated cell-cell contacts. Expression of N-cadherin-deleted proteins lacking all or a portion of the cytoplasmic domain and pharmacological inhibition of actin polymerization, RhoA, and ROCK, were used to determine whether filamentous (F) actin and actomyosin contractility were involved in α3β4 nAChRs localization on the cell membrane.

## Materials and Methods

### Cell Cultures and Transfections

Chinese hamster ovary (CHO) K1 cells were obtained from American Type Culture Collection (ATCC) (Manassas, VA, USA) (ATCC CCL-61) and cultured and handled according to ATCC recommendations. Briefly, CHO cells were grown in plastic tissue culture bottles in high-glucose Dulbecco’s Modified Eagles Medium (DMEM) (Gibco, Invitrogen, Carlsbad, CA, USA) supplemented with 2 mM L-glutamine and 10% fetal bovine serum (FBS) form ATCC. Human colorectal adenocarcinoma Caco-2 cells were obtained from ATCC (ATCC HTB-37) and cultured in plastic tissue culture bottles in Eagle’s Minimum Essential Medium (EMEM) from ATCC supplemented with 10% FBS. Human Embryonic Kidney (HEK) 293 cells were obtained from ATCC (ATCC CRL-1573) and grown in plastic tissue culture bottles in DMEM supplemented with 10% FBS. CHO and Caco-2 cells were trypsinized and replated on 12 mm glass coverslips coated with poly-L-ornithine (Sigma P4857) (Sigma, St Louis, MO, USA) and cultured in 24-well plastic tissue culture plates. When cells reached ∼80% confluence (24 to 48 h after replating), cells were transfected with the desired combination of expression vectors using Lipofectamine 2000 (Invitrogen, Carlsbad, CA, USA) according to the manufacturer’s instructions.

### Antibodies and Reagents

The antibodies used in this study included: mouse monoclonal anti-myc-tag epitope (MEQKLISEEDLNE) (clone 9B11, Cell Signaling Technologies, Danvers, MA, USA); mouse monoclonal anti-human N-cadherin cytoplasmic domain (clone 32/N-cadherin, BD Biosciences 610920**,** San Jose, CA, USA); rat monoclonal anti-chicken N-cadherin extracellular domain (clone NCD2, Invitrogen, Zymed 13-2100); mouse monoclonal anti-polycephalum myxamoebae β-tubulin (clone KMX-1, Chemicon 3408, EMD Millipore Corporation, Billerica, MA, USA); mouse monoclonal anti-sea urchin acetylated α-tubulin (clone 6-11B-1, Sigma T6793); mouse monoclonal anti-Arp3 (clone 4, BD Biosciences 612134); mouse monoclonal anti-human interleukin 2 (IL2) αreceptor (R) subunit extracellular domain (clone MEM-181, Abcam 8235, Cambridge, MA, USA); rabbit polyclonal anti-α-catenin (Invitrogen 71–1200); mouse monoclonal anti-enhanced green fluorescent (EGFP) (Roche 1814460, Indianapolis, IN, USA). Horseradish peroxidase, Cy3, and Cy2 conjugated secondary antibodies were obtained from Jackson ImmunoResearch, West Grove, PA. Alexa-488, Alexa-546, and Alexa-635 conjugated secondary antibodies were purchased from Invitrogen. Reagents: Alexa Fluor 488 Phalloidin (Molecular Probes, Invitrogen); cell permeable C3 transferase (RhoA Inhibitor I Cytoskeleton CT04, Denver, CO); Rho associated kinase (ROCK) inhibitor Y27632 (Cytoskeleton CN06); latrunculin A (LAT-A) (Sigma L5163); cytochalasin D (CTCH-D) (Sigma C8273); dimethyl sulfoxide (DMSO) (Sigma D2650); acetylcholine (Sigma A2661); and epibatidine (Sigma E1145).

### DNA Constructs and Expression Vectors

Neuronal nAChR subunits α3 (CHRNA3) and β4 (CHRNB4) cloned into a pcDNA3.1 expression vector (Invitrogen) were a gift from J. A. Stitzel. To generate α3 and β4 nAChR subunits C-terminally fused to a myc-tag epitope (α3-myc and β4-myc nAChR respectively) [32], the receptor subunits coding sequence excluding the stop codon were PCR amplified and the products cloned into a myc-tagging vector that was generated by replacing the EGFP coding sequence from the pEGFP-N1 vector (Clontech, Palo Alto, CA, USA) with six repeats of the myc-tag epitope sequence (MEQKLISEEDLNE). N-cadherin C-terminally fused to EGFP (N-cadherin-EGFP) was constructed by cloning the protein coding sequence without the stop codon into a pEGFP-N1 vector. Full length chicken N-cadherin cDNA (X07277) in pBluescript [33] (a gift from M. Takeichi) was digested with Apa I, which cut the multiple cloning site region of the vector upstream of N-cadherin and at position 2739 of N-cadherin open reading frame removing the last 3′ end 47 bp including the stop. A double-stranded 65 bp DNA linker was generated having 5′ Apa I and 3′ Age I compatible ends and containing the last 44 bp of N-cadherin plus 21 bp encoding a linker between N-cadherin and EGFP. pEGFP-N1 digested with Apa I and Age I was used for ligating the digested N-cadherin and the dsDNA linker. The ligation generated an expression vector encoding full length N-cadherin C-terminally fused to EGFP with a spacer (SGGSGGPPVAT) between the C-terminal end of N-cadherin and the EGFP start codon. Chicken N-cadherin deletion constructs lacking the last C-terminal 35 amino acids corresponding to the β-catenin binding region (N-cadherin-Δ-β-catenin) or lacking the last C-terminal 157 amino acids corresponding to the entire cytoplasmic domain (N-cadherin-Δ-cyto) were a gift from R. M. Mège [34]. Both N-cadherin deletion constructs were C-terminally fused to EGFP in a pEGFP-N1 vector. A pcDNA3.1 plasmid carrying the red fluorescent protein TagRFP-T [35] was a gift from R. W. Tsien. An expression vector (pcDNA3.1) carrying EGFP with a myristoylation motif was used to label the cell membrane. The sequences of the constructs generated in this study were verified by sequencing analyses. Plasmid DNA used for transfection was generated using endotoxin free Maxiprep Kits (Qiagen, Valencia, CA, USA).

### Western Blot Analysis

CHO and HEK293 cells grown in plastic tissue culture dishes were placed on ice, rinsed with ice-cold Dulbecco’s phosphate buffered saline (D-PBS) containing 1 mM CaCl_2_ and 0.5 mM MgCl_2_ (Gibco), and lysed in protein extraction buffer (25 mM HEPES pH 7.2, 150 mM NaCl, 1% NP40, EDTA-free protein inhibitor cocktail (Roche), 10 mM NaF 1 mM sodium orthovanadate) by pipetting and sonication on ice (10 sec, 2W). Cell lysates were cleared by centrifugation at 14,000 RPM in a microfuge (Eppendorf, Hamburg, Germany) at 4°C. The supernatants were transferred to a new tube and protein concentration determined using the BCA protein assay (Pierce, Thermo Scientific, Rockford, IL, USA). Equal amounts of total proteins from each sample were brought to the same volume in Laemmli buffer [36], boiled, and separated by 10% SDS-PAGE, and proteins electrotransferred to a polyvinylidene difluoride (PVDF) membrane (Millipore, Immobilon-P). Nonspecific binding sites were blocked by incubation for 30 min at room temperature in blocking solution containing 5% non-fat dry milk (Bio-Rad, Hercules, CA, USA) and 0.1% of Tween-20 in Tris-buffered saline (TBS-T). Cell lysates of CHO cells transfected with nAChR subunits were immunoblotted with anti-myc antibodies (9B11). Cell lysates from parental CHO and HEK293 cells were immunoblotted with anti-N-cadherin antibodies (mab 610920). The membranes were washed, incubated with anti-mouse horseradish peroxidase-conjugated secondary antibodies, and developed by chemiluminescence (GE Healthcare, Fairfield, CT, USA). The membrane immunoblotted with N-cadherin antibodies was washed, incubated in stripping solution (100 mM β-mercaptoethanol, 2% SDS, 62.5 mM Tris HCl pH 6.7) for 30 min at 50°C, blocked, immunoblotted with anti-β-tubulin antibodies (mab 3408), washed, incubated with anti-mouse horseradish peroxidase-conjugated secondary antibodies, and developed by chemiluminescence.

### Cell Labeling and Immunostaining

To immunolabel cultured cells grown on glass coverslips, cell culture plates were placed on ice, rinsed with ice-cold D-PBS, fixed with 4% paraformaldehyde in D-PBS for 20 min at room temperature, washed with D-PBS, and blocked with 5% donkey serum (Jackson ImmunoResearch) in D-PBS for 30 min at room temperature. α3β4-myc nAChRs and N-cadherin were cell surface labeled with anti-myc-tag epitope (9B11) and with anti-N-cadherin (NCD2) antibodies respectively at room temperature for 1 h with gentle agitation and washed with D-PBS. To label intracellular proteins, the cells were permeabilized with D-PBS containing 0.2% Triton X-100 and 5% donkey serum for 30 min at room temperature and incubated with Alexa-488 phalloidin or with the desired primary antibody overnight at 4°C with gentle rotation. The antibodies were washed in D-PBS and cells incubated with corresponding conjugated secondary antibodies for 2 h at room temperature, washed in D-PBS, and mounted in Prolong Gold mounting medium (Invitrogen).

### Confocal Microscopy and Image Analysis

Cells were imaged with a Nikon C1-SI confocal microscope (Nikon Instruments, Japan) mounted on a Nikon Eclipse 90i up-right microscope using a Plan Apo 60X/1.4 Nikon oil immersion lens. Optical sections were collected at 0.25 and 0.5-µm intervals independently for each color channel with the appropriate combination of laser wavelength and filters using Nikon EZ-C1 software. Pairs of CHO cells having a distinct cell-cell contact area in which N-cadherin-EGFP was accumulated and having contact-free cell membrane were selected for imaging and analysis. Optical sections were scanned through the middle of the cell volume and 1.5-µm thick stacks of images were rendered to a single plane by maximal projection using EZ-C1 Viewer Nikon software, and analyzed with MetaMorph image analysis software (Universal Imaging Corp, Dowingtown, PA). Distances were calibrated, each color channel was separated, and background fluorescence eliminated by setting a low threshold level of detection. Pixel area for each fluorophore was measure in a 2×2 µm region of interest placed at the center of the cell-cell contact area, at each of the cell contact edges, and at four locations over the contact-free cell membrane (2 samples from each cell). The average of the two samples from the edges and from the four samples of the contact-free cell membrane were used for analysis. Data was logged into Excel software (Microsoft, Seattle, WA) and used to calculate the mean and the standard error of the mean (SEM). In addition to sampling the cell membrane with a 2×2 µm region, measurements of pixel density were carried out on the entire cell-cell contact and contact-free cell membrane by drawing a region of interest including the contact-free cell membrane and a region including the contacting area only. The pixel area was normalized to membrane length (µm). The results from this analysis are presented in Figure S1 and indicate that both methods of assessing pixel density yielded the same results. Cells for image analysis were collected from a minimum of two independent experiments. One-way ANOVA analysis and post-hoc Bonferroni tests were carried out using SigmaStat software (Systat Software, Inc. San Jose, CA). Polarized Caco-2 cells were imaged at 0.25-µm intervals throughout the cell volume and sections from the basal and apical regions were deconvoluted in MetaMorph software and used for display.

### Electrophysiology

CHO cells were cotransfected with α3-myc, β4-myc, and TagRFP-T. Forty-eight hours after transfection, a coverslip of transfected CHO cells was placed in a recording chamber containing extracellular recording solution (NaCl, 150 mM; KCl, 5 mM; CaCl_2_, 2.5 mM, HEPES, 10 mM; and glucose, 150 mM) and mounted on an upright Zeiss Axioskop 2FS Plus microscope (Zeiss, Oberkochen, Germany). Cells were observed with a Zeiss Achroplan 40X/NA0.8 water immersion lens under differential interference contrast (DIC) and fluorescence light illumination. TagRFP-T-expressing CHO cells were selected for recording. Three to 6 MΩ pipette electrodes were filled with intracellular solution (CsCl, 140 mM; HEPES, 10 mM; EGTA, 0.6 mM; and MgATP, 2 mM). After a seal of ≥1 GΩ was obtained, cells were voltage-clamped in whole-cell mode and maintained at −70 mV using a Multiclamp 700A amplifier (Axon Instruments, Sunnyvale, CA, USA), connected to a Digi-Data 1322A analogue-to-digital converter (Digi-Data, Broomfield, CO, USA). Extracellular solution containing acetylcholine (5 mM) or epibatidine (50 µM) was loaded into 1–2 µm tip diameter glass pipette and placed in close proximity to the patched cell. Light positive pressure using a syringe was sufficient to deliver acetylcholine or epibatidine through the pipette. Solutions were held at room temperature (20–22°C) during recordings.

## Results

### α3 and β4 myc-tagged nAChR Subunits Form Functional Cholinergic Channels in CHO Cells

**Figure 1:**
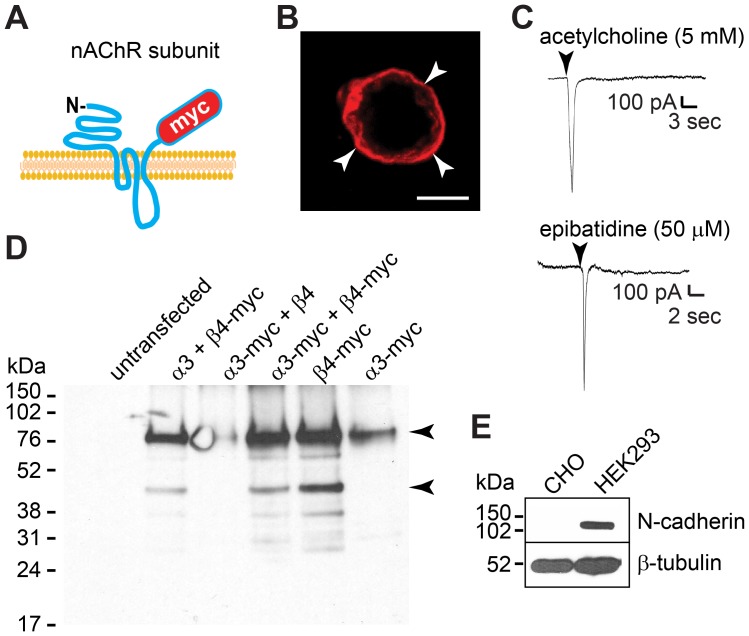
α3-myc and β4-myc nAChR subunits form functional ligand-activated ion channels in CHO cells. A) Schematic drawing of nAChR subunits C-terminally fused to a 6X-myc tag localized to the extracellular side of the cell membrane. B) Confocal image of a CHO cell transfected with α3-myc and β4-myc and cell surface labeled with anti-myc antibodies shows expression of α3β4-myc nAChRs on the cell surface (arrowheads). C) Whole-cell voltage-clamp recordings of CHO cells transfected with α3-myc and β4-myc nAChR subunits. Inward currents were elicited in response to localized application of acetylcholine (5 mM) or epibatidine (50 µM) (arrowheads) through a pipette placed in the proximity of the patched cell indicating expression of functional α3β4 nAChRs on the cell surface. D) Western blot analysis of α3-myc and β4-myc expression in CHO cells cotransfected with tagged and untagged subunits and immunoblotted with anti-myc antibodies. A ∼76 kDa band was detected with both myc-tagged subunits, and a smaller band of ∼40 kDa was detected only in cells transfected with the β4-myc subunit (arrowheads). E) Western blot analysis of parental CHO and HEK293 cell lysates immunoblotted with anti-N-cadherin (32/N-cadherin), stripped, and blotted with β-tubulin antibodies indicates the absence of N-cadherin expression in parental CHO cells. Scale bar in B, 5 µm.

To study the localization of α3β4 nAChRs on the cell surface, both receptors subunits were C-terminally fused to a 6X-myc tag that localizes to the extracellular side of the membrane (Figure 1A). The predicted molecular weights for the α3-myc and β4-myc proteins were 70016 Da and 67559 Da respectively. Western blot analysis with anti-myc antibodies of CHO cell lysates transfected with combinations of untagged and myc-tagged α3 and β4 subunits showed a band of ∼76 kDa in cells expressing α3-myc and β4-myc, corresponding to the size of the full-length protein (Figure 1D). A smaller band of ∼40 kDa was observed in cells transfected with the β4-myc subunit. Although the nature of this smaller fragment has not been characterized, it is likely to be a cleaved product of the β4-myc subunit lacking the N-terminal extracellular domain. Thus, both nAChR subunits were expressed as full length proteins in CHO cells, and their expression did not appear to be affected by the C-terminal fusion of a 6X-myc tag.

Cell surface labeling with anti-myc-tag antibodies of CHO cells cotransfected with α3-myc and β4-myc nAChR subunits, showed expression of the proteins on the cell surface, indicating that this subunit combination is transported to the cell membrane in CHO cells (Figure 1B). CHO cells transfected with only one receptor subunit (α3-myc or β4-myc) showed very low levels of myc immunolabeling on the cell surface (data not shown), indicating that the majority of nAChR subunits expressed on the cell membrane were α3-myc and β4-myc nAChRs heteromers. To determine whether α3-myc and β4-myc nAChR subunits formed functional cholinergic channels on the cell membrane, CHO cells transfected with both subunits were patch-clamped and used to measure whole-cell currents under voltage-clamp configuration. Pressure application of acetylcholine (5 mM) or epibatidine (50 µM) applied through a pipette placed in close proximity to a patch-clamped cell elicited inward currents of ∼1 nA, demonstrating that α3-myc and β4-myc nAChR subunits formed functional ligand-activated ion channels (here called α3β4-myc nAChRs) when expressed on the cell membrane of CHO cells (Figure 1C).

### Formation of N-cadherin-mediated Cell-cell Contacts Regulate α3β4-myc nAChRs Localization on the Cell Surface

**Figure 2:**
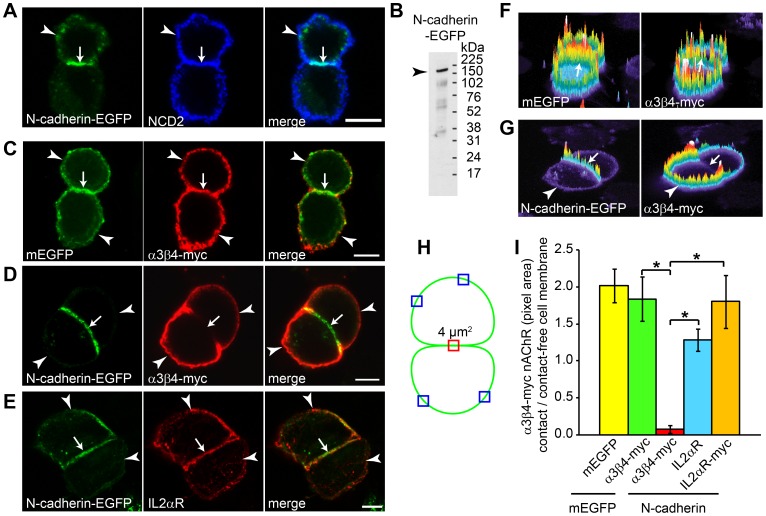
N-cadherin homotypic binding regulates the cell surface localization of α3β4-myc nAChRs. A) CHO cells transfected with N-cadherin-EGFP and cell surface labeled with anti-N-cadherin antibodies (NCD2) show expression of N-cadherin at the cell membrane (arrowheads) and N-cadherin accumulation at the intercellular junction (arrows). B) Western blot analysis of N-cadherin-EGFP expression in CHO cells immunoblotted with anti-N-cadherin antibodies (NCD2). C) CHO cells expressing α3β4-myc nAChRs and mEGFP. Cell surface labeling with anti-myc antibodies shows localization of α3β4-myc nAChRs on both contacting (arrows) and contact-free cell membranes (arrowheads). D) CHO cells expressing α3β4-myc nAChRs and N-cadherin-EGFP show exclusion of α3β4-myc nAChRs from the N-cadherin-mediated cell-cell contact (arrows), while nAChRs are expressed on the contact-free cell membrane (arrowheads). E) CHO cells expressing N-cadherin-EGFP and IL2αR and surface labeled with anti-IL2αR antibodies show localization of IL2αR within the cell-cell contact zone (arrows) and on the contact-free cell membranes (arrowheads). F, G) Fluorescence intensity profiles of the cells shown in panels C and D respectively. Panel F shows localization of both, mEGFP and α3β4-myc nAChRs on the cell surface including the contacting cell membranes (arrows). Panel G shows no expression of α3β4-myc nAChRs at the N-cadherin-mediated cell-cell contact (arrows) and expression on the contact-free cell membrane (arrowheads). H) Schematic drawing of two contacting CHO cells, the red square shows the localization of a 2×2 µm region used for measuring pixel density within the cell-cell contact, and the blue squares show the approximate localization of sampling regions used to measure pixel density on the contact-free cell membrane. I) Analysis of the ratio between cell-cell contacts and contact-free cell membrane of mEGFP, α3β4-myc nAChRs, and IL2αR in CHO cells expressing mEGFP or N-cadherin-EGFP. Values represent the mean ± SEM of each experimental group (mEGFP and α3β4-myc, n = 24; N-cadherin and α3β4-myc, n = 22; N-cadherin and IL2αR labeled with anti-IL2αR antibodies, n = 22; N-cadherin and IL2αR labeled with anti-myc antibodies, n = 28). One-way ANOVA, p<0.001; *post-hoc Bonferroni test comparisons with mEGFP, p<0.05; Bars with an asterisk indicate post-hoc Bonferroni test comparison between the two indicated groups, p<0.05.

To examine whether N-cadherin-mediated cell-cell junctions regulate the localization of α3β4-myc nAChRs on the cell surface, CHO cells were cotransfected with α3β4-myc and N-cadherin C-terminally fused to EGFP. CHO cells transfected with α3β4-myc and mEGFP were used as controls. CHO cells were chosen for these experiments because this cell line does not express N-cadherin endogenously (Figure 1E) [32], and therefore changes in nAChRs distribution could be attributed to the heterologous expression of N-cadherin. CHO cells expressing N-cadherin formed intercellular junctions in which N-cadherin became concentrated at the area of contact between the two cells (Figure 2A). Surface labeling with antibodies against N-cadherin extracellular domain showed a similar distribution of the protein as the one observed with EGFP; however, the sensitivity of detection of N-cadherin with antibody labeling was higher than EGFP fluorescence and it was used for quantifying the density of N-cadherin on the cell membrane. Western blot analysis with anti-N-cadherin antibodies of N-cadherin-EGFP transfected CHO cell lysates demonstrated expression of full length protein (Figure 2B).

The cell surface distribution of α3β4-myc nAChRs was examined in contacting CHO cells co-expressing mEGFP or N-cadherin-EGFP and cell surface labeled with anti-myc antibodies. Pairs of CHO cells having a distinct cell-cell contact region and contact-free cell membrane were used for analysis. In cells expressing mEGFP and α3β4-myc nAChRs, mEGFP appeared evenly distributed on the cell surface including the contact-free cell membrane as well as the area of contact between the two cells (Figure 2C). Similarly to mEGFP, α3β4-myc nAChRs were also distributed over the entire cell surface including the area of contact between cells and the contact-free cell membrane. The localization of mEGFP and α3β4-myc nAChRs on the different regions of the cell surface is illustrated in the fluorescence intensity diagrams shown in Figure 2F. To quantify the α3β4-myc nAChRs density over different areas of the cell membrane, the pixel area of mEGFP and α3β4-myc was measured in a 2×2 µm square placed at the center of the cell-cell contact and at four random locations of the contact-free cell membrane (two on each cell) (Figure 2H). The average of the four measurements from the contact-free cell membrane was used for analysis and the results expressed as the ratio between the pixel area within the cell-cell contact over the contact-free cell membrane. A ratio of ∼2 was found for mEGFP pixel area, indicating that the protein density was ∼2-fold higher within the cell-cell contact (Figure 2I). As the area of cell-cell contact contains two cell membranes, the measurements indicate the expected even distribution of mEGFP on the entire cell surface. Similarly to mEGFP, α3β4-myc pixel area within the cell-cell contact was ∼2-fold higher than the contact-free cell membrane, also indicating that α3β4-myc nAChRs tended to distribute over the entire cell surface (Figure 2I). In contrast, N-cadherin-mediated cell-cell contacts appeared depleted of α3β4-myc nAChRs, while expression of nAChRs on the contact-free cell membrane was unaffected (Figure 2D). Similar results were obtained by measuring the pixel density over the entire cell surface and correcting by membrane length (Figure S1). Expression of N-cadherin within the cell-cell contact area and the absence of α3β4-myc nAChRs within the area of cell-cell contact are illustrated in the fluorescence intensity diagrams shown in Figure 2G. Analysis of the pixel density of α3β4-myc nAChRs showed very low expression levels of nAChRs within N-cadherin-mediated cell-cell contacts while nAChRs density at the contact-free cell membrane was similar to the one observed in CHO cells expressing mEGFP (mEGFP, 210.9±15.6 (n = 24) and N-cadherin, 234.4±20.3 (n = 22) pixel area/4 µm^2^ membrane) (Figure 2I).

To determine whether formation of N-cadherin-mediated cell-cell contacts specifically regulated α3β4-myc nAChRs distribution on the cell surface, CHO cells were cotransfected with N-cadherin and with a plasmid expressing the human IL2αR subunit C-terminally fused to a 6X-myc tag that localizes to the intracellular side of the cell membrane. Transfected CHO cells were fixed and cell surface labeled with anti-IL2αR antibodies (Figure 2E), or permeabilized and immunolabeled with anti-myc antibodies. Analysis of IL2αR pixel area within N-cadherin-mediated cell-cell contacts normalized to the contact-free cell membrane showed a ∼1.5-fold and a ∼2-fold higher receptor density within the cell-cell contact area when the receptors were labeled with anti-IL2αR or anti-myc antibodies respectively, indicating that IL2αR was expressed within the N-cadherin-mediated cell-cell contact zone. These results indicate that formation of N-cadherin-mediated cell-cell contacts regulates α3β4-myc nAChRs distribution on the cell surface by precluding the localization of the receptor within the N-cadherin-mediated intercellular junction.

### N-cadherin Cytoplasmic Domain Regulates α3β4-myc nAChRs Localization at Cell-cell Contacts

**Figure 3:**
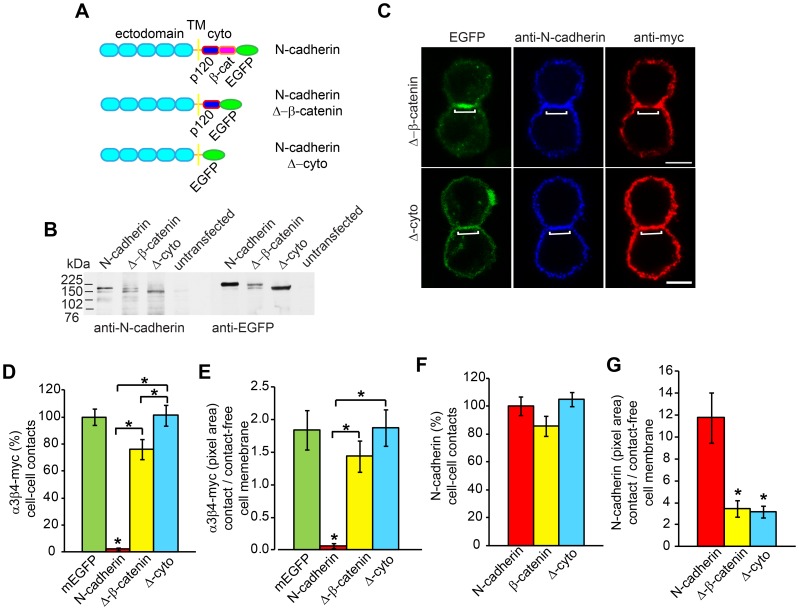
N-cadherin cytoplasmic domain is required to regulate α3β4-myc nAChRs localization on the cell surface. A) Schematic drawings of the N-cadherin constructs used in this study. Full length N-cadherin (N-cadherin) comprised of the ectodomain, the transmembrane domain (TM), and the cytoplasmic domain (cyto). N-cadherin with a deleted β-catenin (β-cat) binding region (N-cadherin-Δ-β-catenin), and N-cadherin with a deletion of the entire cytoplasmic domain (N-cadherin-Δ-cyto) lacks both the p120-catenin (p120) and α-catenin binding regions. The three constructs were C-terminally fused to EGFP. B) Western blot analysis of expression of full length N-cadherin and N-cadherin-deleted proteins in CHO cell homogenates immunoblotted with anti-N-cadherin (NCD2) and anti-EGFP antibodies. C) Confocal images of CHO cells expressing N-cadherin-Δ-β-catenin or N-cadherin-Δ-cyto and α3β4-myc nAChRs. Cells were cell surface labeled with anti-N-cadherin (NCD2) and anti-myc antibodies (9B11). Brackets indicate the cell-cell contact area. D) Analysis of pixel density of α3β4-myc nAChRs within the cell-cell contact area in CHO cells expressing mEGFP (n = 24), N-cadherin (n = 22), N-cadherin-Δ-β-catenin (n = 25), or N-cadherin-Δ-cyto (n = 27). α3β4-myc nAChRs pixel density within the cell-cell contact area between cells expressing mEGFP was considered 100%. E) Ratio of α3β4-myc nAChRs pixel density between the cell-cell contact area and the contact-free cell membrane. F) Analysis of the expression levels of N-cadherin-deleted constructs, N-cadherin-Δ-β-catenin (n = 25) and N-cadherin-Δ-cyto (n = 27), at the cell-cell contact membrane compared to the expression levels of N-cadherin (100%) (n = 22). G) Ratio of pixel density between the cell-cell contact and the contact-free cell membrane for N-cadherin, N-cadherin-Δ-β-catenin, and N-cadherin-Δ-cyto deleted constructs immunolabeled with anti-N-cadherin antibodies (NCD2). Values in D, E, F and G represent the mean ± SEM of each experimental group. One-way ANOVA in D, E, and G, p<0.001, and in F, p>0.05; post-hoc Bonferroni test comparisons with mEGFP (D and E) or N-cadherin (G), *p<0.05. Bars with an asterisk indicate post-hoc Bonferroni test comparison between the two indicated groups, p<0.05. Scale bars, 5 µm.

N-cadherin cytoplasmic domain binds β-catenin and p120-catenin, which participate in the interaction of N-cadherin with the actin cytoskeleton and regulate cytoskeletal dynamics downstream of RhoA GTPase [19], [20], [37]. To examine whether the intracellular domain of N-cadherin was required for the localization of α3β4-myc nAChRs outside N-cadherin-mediated cell-cell contacts, CHO cells expressing α3β4-myc AChRs were cotransfected with constructs expressing C-terminally truncated N-cadherin molecules. One of the deleted constructs lacked the β-catenin binding region (N-cadherin-Δ-β-catenin), while the other construct lacked the entire N-cadherin cytoplasmic domain and therefore missing both, the p120-catenin and β-catenin binding sites (N-cadherin-Δ-cyto) (Figure 3A). Both truncated N-cadherin proteins were C-terminally fused to EGFP [34], [38], [39]. Expression of full length and truncated N-cadherin proteins in CHO cells was confirmed by Western blot analysis with anti-N-cadherin extracellular domain and anti-EGFP antibodies (Figure 3B). As observed with the full length protein, both truncated N-cadherins were expressed on the surface of CHO cells and concentrated at the cell-cell contact area, indicating that homotypic binding between N-cadherin extracellular domains was not affected (Figure 3C) [38].

α3β4-myc nAChRs density within the cell-cell contact region of CHO cells expressing N-cadherin truncated proteins was measured as described above and compared to α3β4-myc nAChRs density observed in the cell-cell contact zone between CHO cells expressing mEGFP (Figure 3D). The density of α3β4-myc nAChRs within N-cadherin-mediated cell-cell contacts was ∼2% of the density of α3β4-myc nAChRs observed in contacting CHO cells expressing mEGFP (Figure 3D). In contrast, cell-cell contacts formed by N-cadherin-Δ-β-catenin have a substantially higher concentration of nAChRs (∼76% of control values) (Figure 3D). Furthermore, α3β4-myc nAChRs density in cell-cell contacts mediated by N-cadherin-Δ-cyto was almost identical to the nAChRs density observed in contacting CHO cells expressing mEGFP (Figure 3D). Analysis of the ratio of α3β4-myc nAChRs density between cell-cell contacts and contact-free cell membrane showed that α3β4-myc nAChRs density was higher on the contact-free cell membrane in cells expressing N-cadherin. In contrast, the ratio was closer to control levels in cells expressing N-cadherin-Δ-β-catenin, and in N-cadherin-Δ-cyto expressing cells the ratio was undistinguishable from controls (Figure 3E). Therefore, these results indicated that the cytoplasmic domain of N-cadherin was necessary for preventing the localization of α3β4-myc nAChRs within the cell-cell contact or it was required for promoting the localization of α3β4-myc nAChRs to areas of the cell surface outside the cell-cell contact zone.

Binding of p120-catenin to type I cadherins cytoplasmic domain regulates cadherin turnover by masking an endocytotic signal [40]–[42] and modulates the stability of cadherin-mediated cell junctions. However, while deletion of the β-catenin binding site of E-cadherin disrupts cell aggregation, removal of the entire E-cadherin cytoplasmic domain does not, suggesting that formation of N-cadherin mediated cell-cell contacts were not affected in cells expressing N-cadherin-Δ-cyto [43]. To determine whether the increase in α3β4-myc nAChRs localization within the cell-cell contact area in cells expressing N-cadherin-deleted proteins was due to a decrease in the concentration of N-cadherin at cell-cell contacts, the pixel density of N-cadherin-Δ-β-catenin and N-cadherin-Δ-cyto within the cell-cell contact zone was compared with the pixel density in cell-cell contacts mediated by N-cadherin. This analysis showed that the expression levels of both truncated N-cadherins were similarly to the ones observed with the full length protein (Figure 3F). Analysis of the cell-cell contact size (Figure S2) and of the cell surface expression levels of α3β4-myc nAChRs (Figure S3) showed that cell-cell contact formation and nAChRs expression were not affected by expression of truncated N-cadherin proteins. This indicates that the ability of N-cadherin to establish homotypic binding between ectodomains on apposed cell membranes was not disrupted by deletions of N-cadherin cytoplasmic domain, even though the adhesive strength of the cell-cell contact is impaired by the loss of the β-catenin binding site [43]. The ratio of N-cadherin density between the cell-cell contact zone and the contact-free cell membrane was significantly lower in cells expressing N-cadherin-deleted proteins (Figure 3G), indicating that the loss of N-cadherin interaction with the cytoskeleton resulted in a more even distribution of the protein on the cell surface. Thus, the increase of α3β4-myc nAChRs expression within the cell-cell contact zone was not due to the loss of N-cadherin at the contact site but rather due to regulatory functions of N-cadherin cytoplasmic domain.

### Regulation of α3β4-myc nAChRs Localization Involves RhoA and ROCK

**Figure 4:**
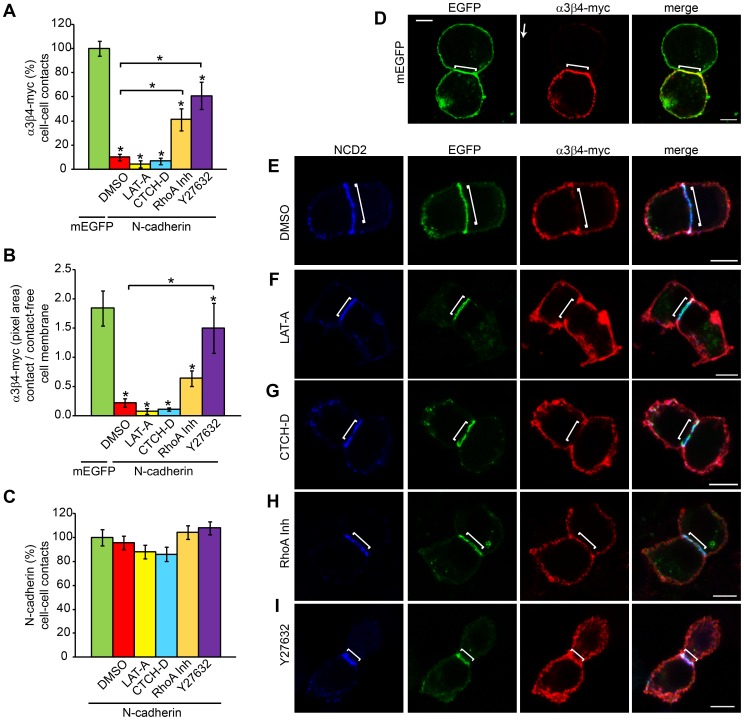
ROCK regulates N-cadherin-mediate α3β4-myc nAChRs distribution on the cell surface. CHO cells expressing N-cadherin and α3β4-myc nAChRs were treated with DMSO (10 µl/ml) or with the indicated drugs: LAT-A 10 µM, CTCH-D 2 µM, RhoA Inh 1 µg/ml, and the ROCK inhibitor Y27632 10 µM. A) Effect of pharmacological treatments on α3β4-myc nAChRs density at N-cadherin-mediated cell-cell contacts as compared to cell-cell contacts between untreated cells expressing mEGFP and α3β4-myc nAChRs (100%) (mEGFP, n = 24; DMSO, n = 20; LAT-A, n = 21; CTCH-D, n = 15; RhoA Inh, n = 21; and Y27632, n = 21). B) Ratio between α3β4-myc nAChRs pixel area within cell-cell contacts and contact-free cell membrane in N-cadherin-mediated cell-cell contacts treated with the indicated drugs as compared to cell-cell contacts between cells expressing mEGFP (100%). C) N-cadherin pixel area within the cell-cell contact compared to untreated cells (100%). Values represent the mean ± SEM of each experimental group. One-way ANOVA in A and B, p<0.001, and in C, p>0.05; post-hoc Bonferroni test comparisons with mEGFP transfected cells (A and B) or untreated cells (C). Bars in A and B indicate comparisons of drug-treated cells against DMSO-treated cells, *p<0.05. D - I) Representative confocal images of CHO cells expressing α3β4-myc nAChRs and mEGFP (D) or N-cadherin-EGFP (E, F, G, H, and I) and treated with the indicated drug. Cells were surface labeled with anti-myc (9B11) and anti-N-cadherin (NCD2) antibodies. Bracket indicates the area of contact between cells. Scale bar, 5 µm.

N-cadherin cytoplasmic domain binds to and regulates the actin cytoskeleton through β-catenin and p120-catenin. Thus, the expression of α3β4-myc nAChRs within cell-cell contacts mediated by N-cadherin molecules with all or a portion of the cytoplasmic domain deleted, suggested that the actin cytoskeleton was involved in the regulation of nAChRs distribution on the cell surface. To examine whether actin polymerization was required to maintain α3β4 nAChRs outside the cell-cell contact zone, the effect of inhibitors of actin polymerization on the cell surface distribution of α3β4-myc nAChRs was analyzed in CHO cells expressing N-cadherin. Transfected CHO cells were treated with LAT-A or with CTCH-D for 4h, fixed, immunostained, and α3β4-myc nAChRs density within the cell-cell contact area and on the contact-free cell membrane was calculated as described above. CHO cells treated with DMSO were used as controls and the results were compared to the density of α3β4-myc nAChRs in mEGFP expressing cells (100%). LAT-A and CTCH-D both block actin polymerization by sequestering G-actin or by capping the barbed ends of F-actin respectively [44], [45]. Both drugs caused a substantial disruption of the actin cytoskeleton as determined by changes in the distribution of cortical F-actin labeled with phalloidin (data not shown); however, N-cadherin density at cell-cell junctions was not affected (Figure 4C). α3β4-myc nAChRs density at N-cadherin-mediated cell-cell contacts in cells treated with LAT-A or CTCH-D remained very low and statistically undistinguishable to the density of α3β4-myc nAChRs observed in DMSO treated cells (Figure 4A). Furthermore, the ratio between nAChRs density in the cell-cell contacts and the contact-free cell membrane remained well below one, indicating that the receptors were primarily localized to the contact-free cell membrane (Figure 4B).

Cadherins homotypic binding regulates Rho GTPases, which participate in the formation and expansion of cadherin-mediated cell-cell contacts [46]. Therefore, it was possible that N-cadherin-mediated regulation of the actin cytoskeleton via RhoA and its downstream effector Rho-associated protein kinase (ROCK), participated in the redistribution of α3β4-myc nAChRs on the cell surface. To test this hypothesis, the effect of inhibiting RhoA and ROCK activity on the density of α3β4-myc nAChRs on the cell surface was examined by treating CHO cells expressing N-cadherin and α3β4-myc nAChRs with a cell permeable RhoA inhibitor (C3 transferase) and with the ROCK inhibitor Y27632. Analysis of α3β4-myc nAChRs density in CHO cells treated with the RhoA inhibitor showed a significant increase in α3β4-myc nAChRs concentration at the cell-cell contact zone as compared to CHO cells treated with DMSO (Figure 4A). While DMSO-treated cells showed ∼10% of α3β4-myc nAChRs density within N-cadherin mediated cell-cell contacts as compared to contacts formed between mEGFP expressing CHO cells, cells treated with the RhoA inhibitor showed ∼41% of α3β4-myc nAChRs density within the contact zone. Although, α3β4-myc nAChRs density was significantly lower than the density observed in mEGFP transfected cells, the ability on N-cadherin homotypic binding to exclude α3β4-myc nAChRs from the cell-cell contact area was significantly diminished (Figure 4A). The ratio between nAChRs density in the cell-cell contact zone and the contact-free cell membrane was substantially increased, although it remained below one (Figure 4B). Inhibition of ROCK by Y27632 caused an even higher increase in α3β4-myc nAChRs density within N-cadherin mediated cell-cell contacts. nAChRs density was ∼60% of the density detected in mEGFP transfected cells and it was significantly higher to the density of α3β4-myc nAChRs detected in N-cadherin-transfected cells treated with DMSO (Figure 4A). Furthermore, the ratio of α3β4-myc nAChRs density between the cell-cell contact and the contact-free cell membranes was above one and significantly higher that the ratio observed in DMSO-treated cells (Figure 4B). Inhibition of RhoA and ROCK did not affect the density of N-cadherin within the cell-cell contact area (Figure 4C) indicating that the increase in α3β4-myc nAChRs density within the cell-cell contact zone was not due to the loss of N-cadherin at the contact site.

**Figure 5:**
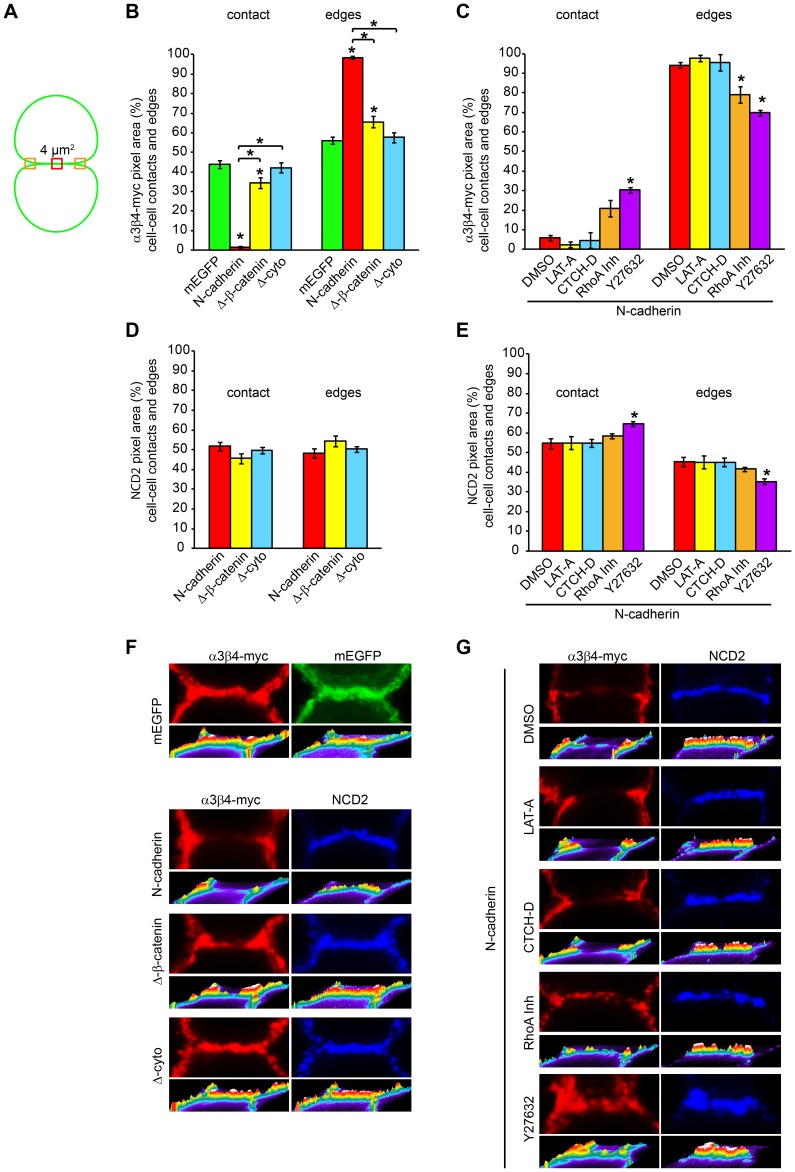
N-cadherin homotypic binding promotes α3β4-myc nAChRs localization at the edges of the cell-cell contact. A) Schematic drawing of two contacting cells showing the areas of the cell surface in which α3β4-myc nAChRs and N-cadherin pixel area was measured in 2×2 µm regions placed at the center (red) and at each of the edges of the cell-cell contact (orange). The average of the two measurements of the edges was used for analysis, and the sum of the measurements at the center and edges at the cell-cell contact was considered 100%. B) Analysis of the percentage of α3β4-myc nAChRs pixel area distribution between the center and the edges of cell-cell contacts in cell junctions mediated by mEGFP (n = 24), N-cadherin (n = 22), N-cadherin-Δ-β-catenin (n = 25), and N-cadherin-Δ-cyto (n = 27). C) Analysis of the effect of inhibitors of actin polymerization (LAT-A 10 µM, n = 21; and CTCH-D 2 µM, n = 16), RhoA (RhoA Inh 1 µg/ml, n = 21), and ROCK (Y27632 10 µM, n = 21) on α3β4-myc nAChRs density at the contact and edges of N-cadherin-mediated cell-cell contacts. Cells treated with DMSO (10 µl/ml, n = 20) were used as controls. D) Analysis of the percentage distribution of N-cadherin and N-cadherin-deleted proteins between the cell-cell contact and the edges of contact zone. E) Analysis of the effect of DMSO, LAT-A, CTCH-D, RhoA Inh, and Y27632 on N-cadherin density at the contact zone and at the edges of cell-cell contacts. Values in B, C, D, and E represent the mean ± SEM of each experimental group. One-way ANOVA of pixel density in cell-cell contacts and the edges of the contact in B and C, p<0.001; post-hoc Bonferroni test comparisons with cells transfected with mEGFP (B) and cells treated with DMSO (C), *p<0.05. Bars with an asterisk indicate post-hoc Bonferroni test comparison between the two indicated groups, p<0.05. One-way ANOVA in C, p>0.05. One-way ANOVA in D, p<0.001, post-hoc Bonferroni test comparisons with DMSO treated cells, *p<0.05. F and G) Representative confocal images of the cell-cell contact area of CHO cells expressing α3β4-myc nAChRs and mEGFP, N-cadherin, N-cadherin-Δ-β-catenin, or N-cadherin-Δ-cyto (F), and of CHO cells expressing α3β4-myc nAChRs and N-cadherin and treated with the indicated drugs (G). Cells were immunolabeled with anti-myc (9B11) and anti-N-cadherin (NCD2) antibodies. The images show the fluorescence signal (above) and the intensity profile (below) for each fluorophore.

During formation of E-cadherin mediated cell-cell contacts, RhoA and actomyosin contractility are primarily active at the edges of the contacting cell membranes and are necessary for the expansion and maintenance of the intercellular junction [46]. The activation of RhoA at distinct sites of the cell membrane suggests that regulation of the actin cytoskeleton at the edges of N-cadherin-mediated cell-cell contacts may participate in the differential distribution of α3β4-myc nAChRs on the cell surface. To examine α3β4-myc nAChRs density at the edges N-cadherin-mediated cell-cell contacts, the pixel area of α3β4-myc nAChRs within a 2×2 µm square was measured at each of the edges of the cell-cell contacts, and the average was used to calculate the percentage of nAChRs density at the center of the cell-cell contact zone and at the edges of the contact (Figure 5A). As both, the cell-cell contact and the edges of the contact contain two cell membranes, it was expected that the density of α3β4-myc nAChRs at the edges of the contact would be similar to the density of the receptors within the contacting area. In fact, in cells expressing mEGFP, α3β4-myc nAChRs distribution between the contact zone and the edges of the contact was close to the expected 50% (Figure 5B and F). In contrast, in N-cadherin-mediated cell-cell contacts, most of α3β4-myc nAChRs were at the edges of the contact zone (Figure 5B and F). However, the percentage of α3β4-myc nAChRs density at the edges of cell-cell contacts mediated by N-cadherin-Δ-β-catenin was diminished and returned to control levels in cell-cell contacts mediated by N-cadherin-Δ-cyto (Figure 5B and F). Treatment with LAT-A and CTCH-D did not affect the accumulation of α3β4-myc nAChRs at the edges of N-cadherin-mediated cell-cell contacts, which remained similar to the percentage of α3β4-myc nAChRs detected in cells treated with DMSO (Figure 5C and G). In contrast, the percentage of receptor density at the edges of the cell-cell contacts was significantly decreased by treatment with RhoA and ROCK inhibitors (Figure 5C and G). This indicates that N-cadherin-mediated cell-cell contacts drive α3β4-myc nAChRs localization outside the cell-cell contact area and promotes the localization of the receptors at the edges of the contact zone through a mechanism involving N-cadherin cytoplasmic domain and actomyosin contractility. N-cadherin distribution between the cell-cell contact zone and the edges of the contact was found to be even in contacts mediated by N-cadherin and by N-cadherin deleted proteins (Figure 5D), and N-cadherin distribution between the two regions was not affected by inhibitors of actin polymerization and RhoA (Figure 5E). However, a significant decrease in N-cadherin at the edges of the cell-cell contact was found in cells treated with the ROCK inhibitor Y27632, suggesting that actomyosin contractility was required to maintain the concentration of N-cadherin at the edges of the contact zone.

### α3β4-myc nAChRs are Targeted to the Apical Cell Surface and Colocalize with F-actin and Arp3 in Polarized Caco-2 Cells

**Figure 6:**
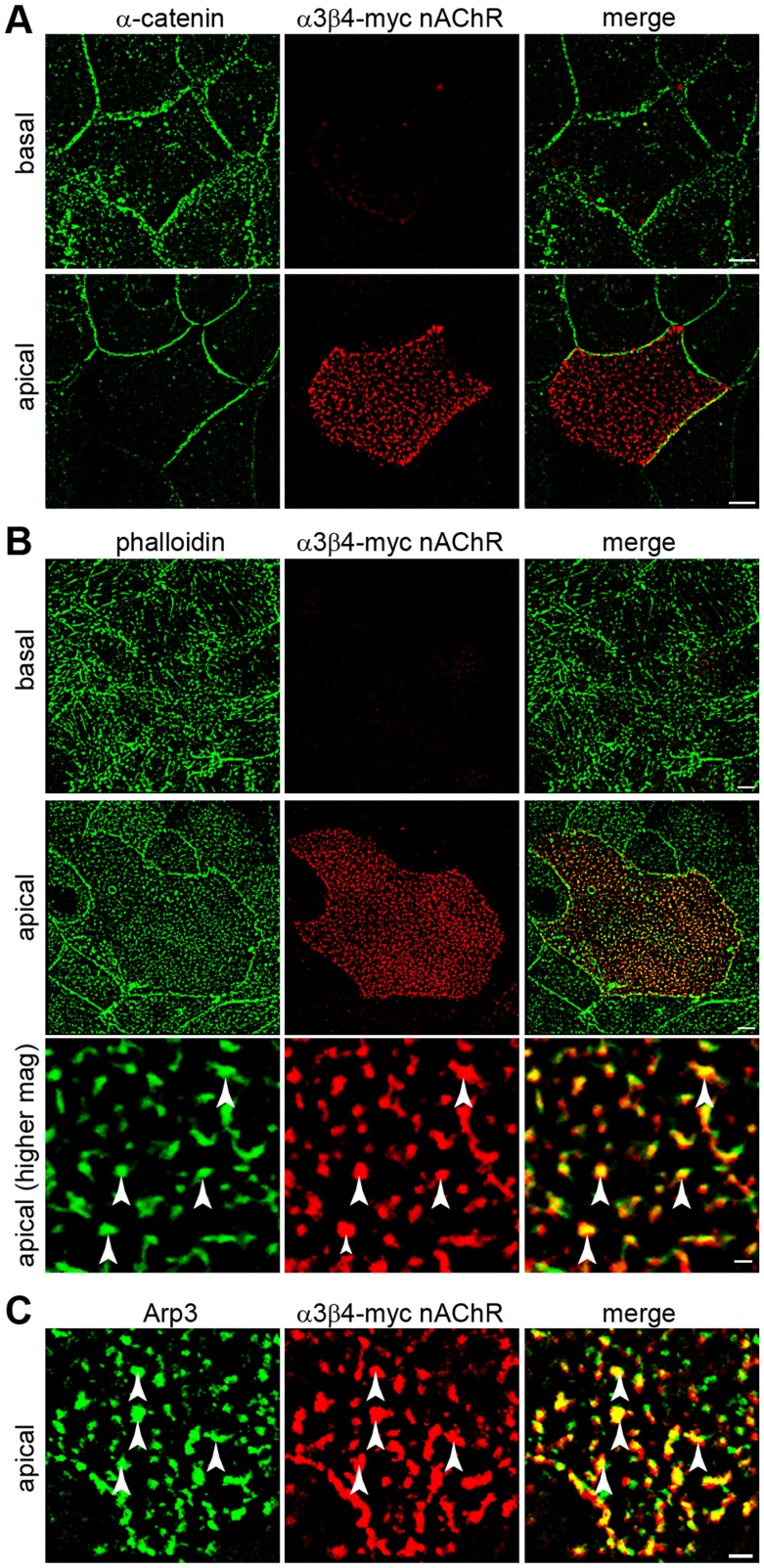
α3β4-myc nAChRs localize to the apical cell surface and colocalize with F-actin and Arp3. Monolayers of Caco-2 cells expressing α3β4-myc nAChRs were fixed, surface labeled with anti-myc antibodies, permeabilized, and colabeled with anti-α-catenin (A), Alexa-488 phalloidin (B), and anti-Arp3 (C) antibodies. A) Deconvoluted confocal images of the basal and apical regions of polarized Caco-2 cells show localization of α-catenin on the basolateral cell membrane while α3β4-myc is exclusively localized to the apical cell surface. B) Deconvoluted confocal images of the basal and apical regions of polarized Caco-2 cells colabeled with Alexa-488 phalloidin show localization of F-actin in both the basolateral and apical cell surface. Higher magnification images of the apical cell surface show colocalization of α3β4-myc nAChRs with F-actin (arrowheads). C) Deconvoluted confocal images of the apical region of Caco-2 cells show colocalization of α3β4-myc nAChRs with Arp3 (arrowheads). Scale bar in A and B, 5 µm; scale bar in B higher magnification and in C, 1 µm.

**Figure 7:**
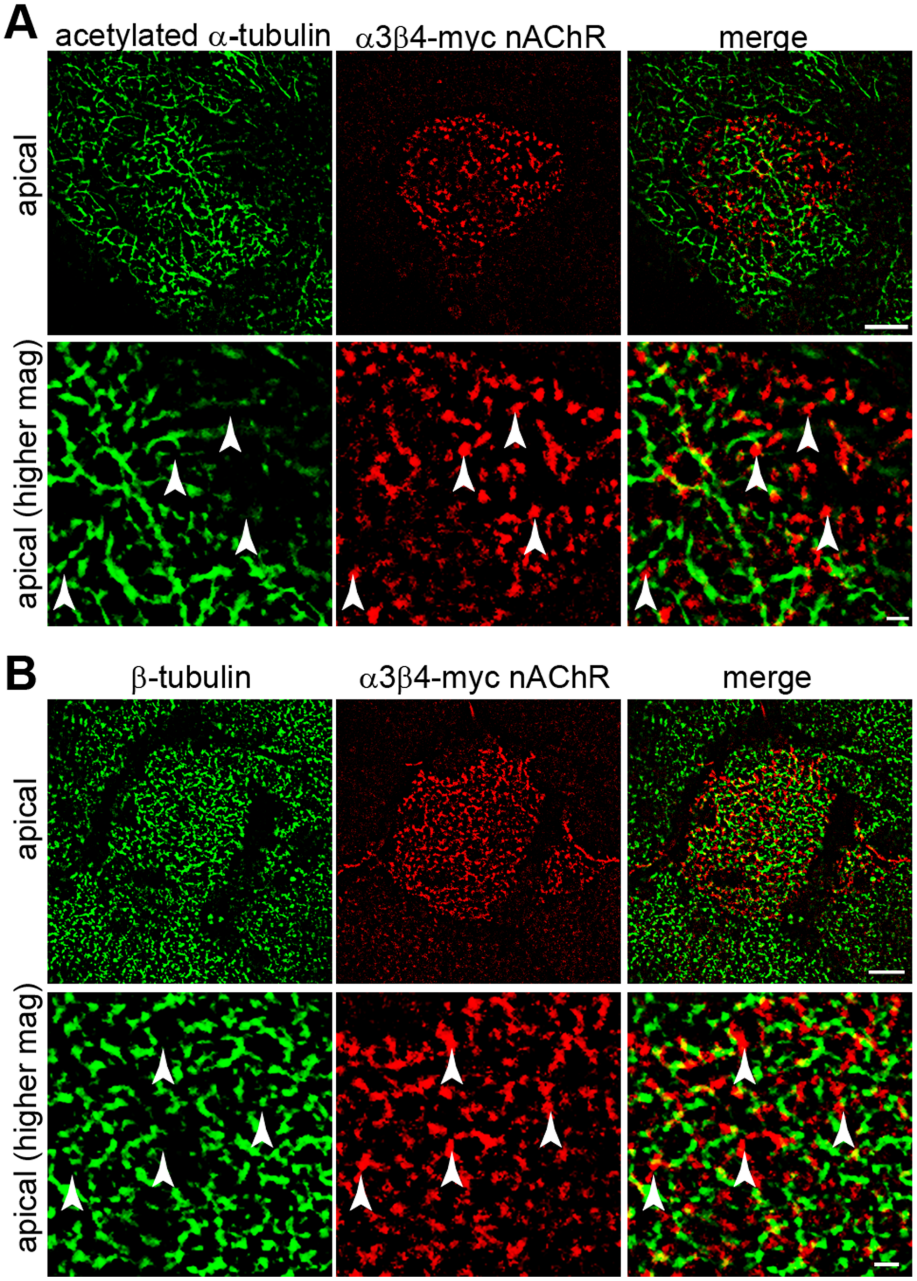
α3β4-myc nAChRs at to the apical cell surface do not colocalize with microtubules. Deconvoluted confocal images at low and high magnification of the apical surface of α3β4-myc nAChRs expressing Caco-2 cells immunolabeled with anti-acetylated α-tubulin (A) and β-tubulin (B) antibodies show no colocalization of α3β4-myc nAChRs with microtubules (arrowheads). Scale bar in lower magnification images (top row in A and B), 5 µm; scale bar in higher magnification images (lower row in A and B), 1 µm.

The results described so far indicate that localization of α3β4-myc nAChRs on the cell surface of CHO cells is regulated by formation of N-cadherin-mediated junctions and requires N-cadherin cytoplasmic domain and actomyosin contractility, suggesting that α3β4-myc nAChRs localization on the cell surface is somehow regulated by the actin cytoskeleton. However, these experiments did not determine whether α3β4-myc nAChRs are targeted to a particular area of the cell surface and associate with F-actin. To examine whether α3β4-myc nAChRs are targeted to a particular area of the cell membrane, polarized Caco-2 cells expressing α3β4-myc nAChRs were surface labeled with anti-myc antibodies. To identify the basolateral and the apical cell membranes, the cells were permeabilized, colabeled with anti-α-catenin antibodies, and confocal images acquired throughout the cell volume. As expected, α-catenin was found at the lateral membrane and was not detected on the apical cell surface (Figure 6A). In contrast, α3β4-myc nAChRs were absent from the basolateral cell membrane and exclusively localized at the apical membrane of the cell. The receptors formed small aggregates (<1 µm in diameter) distributed over the entire apical surface. To examine the distribution of α3β4-myc nAChRs with respect to the actin cytoskeleton, polarized Caco-2 cells expressing α3β4-myc nAChRs were surface labeled with anti-myc antibodies and colabeled with Alexa-488 phalloidin to identify F-actin. In polarized epithelial cells, F-actin forms a distinct cortical cytoskeleton associated with cadherin-mediated intercellular junctions on the lateral membranes as well as actin bundles perpendicular to the basal membrane [47]. In addition, F-actin is localized to the contact-free apical cell surface [48]. Similarly to previous observations, F-actin was found at the basolateral membrane and at the apical cell surface in polarized Caco-2 cells. Analysis of the localization of F-actin and α3β4-myc nAChRs on the apical cell surface showed that the two proteins were colocalized (Figure 6B), indicating that this form of nAChRs localizes to a particular domain of the apical cell membrane enriched in F-actin. Actin polymerization is driven by the actin nucleator complex Arp2/3, and therefore, the localization of Arp3 on the apical cell surface was examined by colabeling Caco-2 cells expressing α3β4-myc nAChRs with anti-Arp3 antibodies. As seen with phalloidin, Arp3 was detected on the apical cell membrane and colocalized with α3β4-myc nAChRs (Figure 6C). Finally, to determine whether the α3β4-myc nAChRs colocalized with microtubules, Caco-2 cells were colabeled with anti-acetylated α-tubulin and with anti-β-tubulin antibodies; however, none of these two microtubule markers appeared associated with α3β4-myc nAChRs (Figure 7). These results indicate that α3β4-myc nAChRs are expressed only on the apical surface of polarized epithelial cells and are associated with areas of the cell membrane enriched in a particular pool of F-actin, which is probably polymerized by the Arp2/3 actin nucleator complex.

## Discussion

Homo and heteromeric neuronal nAChRs expressed throughout the nervous system are localized to synaptic and extra-synaptic sites of the cell membrane [4], [5]. Localization of nAChRs with distinct subunit compositions at particular domains of the cell surface involves receptor targeting, incorporation into membrane microdomains, and association with scaffolding proteins [6], [7], [49]. The present study was aimed at determining whether N-cadherin-mediated cell-cell contacts regulate the cell surface localization of α3β4 nAChRs expressed in CHO cells. The study found that N-cadherin homotypic binding between transfected CHO cells causes the withdrawal of α3β4 nAChRs from the area of cell-cell contact and the accumulation of the receptor at the edges of the contact zone through a mechanism that requires N-cadherin cytoplasmic domain and actomyosin contractility. This suggests that regulation of the actin cytoskeleton directly beneath the N-cadherin-mediated cell-cell contacts and at the margins of the contact zone participates in the localization of α3β4 nAChRs in particular areas of the cell surface. The fact that α3β4 nAChRs colocalize with F-actin and Arp3 at the apical surface of polarized epithelial cells supports the view that direct or indirect interactions of α3β4 nAChRs with a distinct pool of F-actin regulate receptor localization on the cell membrane.

Analysis of *de novo* formation of E-cadherin mediated cell-cell contacts using live-cell imaging in epithelial cells showed that during the first stage of contact formation E-cadherin rapidly accumulates at incipient contact sites and promotes the outward spreading of the intercellular contact through the formation of lamellipodia via a Rac1-dependent mechanism [46], [50]. This first stage in cell-cell contact formation was followed by a second stage involving the expansion of the cadherin-mediated cell-cell contact through a mechanism requiring localized RhoA activation at the edges of the contact zone and actomyosin contractility downstream of ROCK [46]. The growth of the cadherin-mediated cell-cell contact was paralleled by changes in F-actin cortical cytoskeleton, which was removed from the cell-cell contact sites while actin bundles remained present at the edges of the cell-cell contact zone, where active RhoA and phospho myosin II accumulated [46], [51]. The present study looked at N-cadherin-mediated cell-cell contacts in CHO cells 36 to 48 hs after transfection. Although the age of the cell-cell contacts analyzed was unknown, cell-cell contacts were selected by their N-cadherin expression, and therefore, expected to be at the second stage of cell-cell contact formation described above. If the dynamics of small-Rho GTPase activation and of the changes in cortical F-actin distribution during de novo formation of N-cadherin-mediated cell-cell contacts are similar to the ones observed in E-cadherin-mediated contacts, it is expected that RhoA and actomyosin contractility downstream of ROCK were active at the edges of the N-cadherin-mediated cell-cell contacts. Therefore, the exclusion of α3β4 nAChRs from the center of the contact zone and their accumulation at the edges of the cell-cell contact may be driven by activation of RhoA and ROCK at the edges of the contact zone. Indeed, pharmacological inhibition of both RhoA and ROCK presumably removed the boundaries at the periphery of the cell-cell contact area and allowed α3β4 nAChRs to diffuse into the contact zone. Furthermore, exclusion of α3β4 nAChRs from the cell-cell contact zone required the p120-catenin binding domain because expression of N-cadherin lacking the cytoplasmic domain completely abolished the exclusion of nAChRs from the contact zone. Although, the effect of N-cadherin engagement on p120-catenin binding and RhoA activity may depend on the cellular context [52], [53], the present results suggest that the loss of the p120-catenin binding site of N-cadherin cytoplasmic domain inhibited RhoA, presumably by maintain p120-catenin uncoupled from N-cadherin [20], [54], which resulted in the diffusion of the receptors within the cell-cell contact area.

The results discussed above suggest that regulation of F-actin at the edges of N-cadherin-mediated cell-cell contacts downstream of ROCK causes an outward mobilization of α3β4 nAChRs during formation of N-cadherin-mediated cellular junctions and operates as a barrier preventing the diffusion of the α3β4 nAChRs within the cell-cell contact zone once the junction has developed. The colocalization of α3β4 nAChRs with F-actin and Arp3 in the apical surface of polarized cells favors the view that α3β4 nAChRs associate with a distinct pool of F-actin [47], [48], which regulates α3β4 nAChRs localization on the cell membrane. In autonomic ganglia, nAChRs containing the α3 subunit cluster at postsynaptic densities and interact with the actin and microtubule cytoskeleton through a molecular complex organized by adenomatous polyposis coli (APC) [55], [56], indicating that interactions of α3β4 nAChRs with the actin cytoskeleton through adaptor proteins regulate nAChR localization on the cell surface.

Cell-cell contacts mediated by N-cadherin-Δ-β-catenin also showed higher α3β4 nAChRs density as compared to the cell-cell contacts mediated by full length N-cadherin. Although the increase in receptor density was not as significant as the one observed with N-cadherin molecules lacking the entire cytoplasmic domain, the loss of the β-catenin binding site affected the ability of N-cadherin to maintain α3β4 nAChRs excluded from the cell-cell contact zone. Therefore, mechanisms involving the recruitment of β-catenin to the N-cadherin-mediated junction may also contribute to the loss of α3β4 nAChRs from N-cadherin-mediated contacts. As β-catenin participates in the interaction between cadherins and the actin cytoskeleton, it is possible that the regulation of actin contractility downstream ROCK and/or the localization of a particular pool of F-actin at the edges of the contact zone require β-catenin. An alternative mechanism to explain the loss of α3β4 nAChRs from N-cadherin-mediated cell-cell contacts is by the endocytosis of the receptor at the contact zones. For instance, β-catenin binds to scribble, a tumor suppressor factor implicated in epithelial cell polarity and in synapse formation [31], [57], [58]. Scribble binds to β-Pix, a guanine nucleotide exchange factor (GEF) for Rho GTPases [59], and with its partner GIT1, a GTPase-activating protein (GAP) for the ARF family of small GTPases [60]–[62]. Scribble controls the internalization of hormone receptors by regulating ARF6 via the β-Pix-GIT1 complex [61], and activation of ARF6 and Rac1 in CHO cells expressing muscular nAChRs causes nAChRs endocytosis [63], [64]. As both, ARF6 and Rac1, are regulated by the β-Pix-GIT1 complex and its binding to scribble, it possible that recruitment of scribble by β-catenin to N-cadherin-mediated cell-cell contacts induces α3β4 nAChRs endocytosis from the contacting cell membrane.

In conclusion, N-cadherin-mediated cell-cell junctions at cholinergic synaptic contacts may regulate α3β4 nAChRs localization by excluding the receptor from the contact site and/or by promoting the accumulation of the nAChRs in a membrane domain adjacent to the N-cadherin-mediated cell-cell junction. As N-cadherin commonly aggregates into puncta localized adjacent to the active zone and the postsynaptic density, α3β4 nAChRs clustering may occur at the postsynaptic membrane apposed to the active zone or at extrasynaptic sites. It is interesting to note that N-cadherin expression in CHO cells induces presynaptic differentiation in cultured central cholinergic neurons; however, the hemisynaptic sites formed were found incapable of activating α3β4 nAChRs expressed on the CHO cell [32]. This may be due to the inability of N-cadherin to develop neurotransmitter release sites, or because N-cadherin failed to recruit α3β4 nAChRs at the cell-cell contact site. A similar mechanism has been observed with thrombospondins, in which expression of thrombospondin 1 in heterologous cells resulted in formation of functional presynaptic terminals but silent synaptic contacts due to the lack of accumulation of AMPA receptors on the postsynaptic cell membrane [65]. Therefore, N-cadherin homotypic binding appears to regulate α3β4 nAChRs localization on the cell membrane; however, the specific localization of α3β4 nAChRs with respect to the active zone may involve other neuronal components. Nevertheless, the present study showed that N-cadherin homotypic binding in CHO cells plays an important role in the localization of α3β4 nAChRs in a particular area of the cell membrane presumably by regulating actomyosin contractility of a particular pool of F-actin that interacts directly or indirectly with α3β4 nAChRs. Further studies are required to determine whether this mechanism participates in the localization and assembly of α3β4 nAChRs clusters in neurons.

## Supporting Information

Figure S1 **N-cadherin cytoplasmic domain is required to regulate α3β4-myc nAChRs localization on the cell surface.** Pixel area was measured in the entire cell-cell contact membrane and the entire contact-free cell membrane and normalized to membrane length (µm). A) Analysis of pixel area of α3β4-myc nAChRs per µm of cell membrane within the cell-cell contact area in CHO cells expression mEGFP (n = 24), N-cadherin (n = 22), N-cadherin-Δ-β-catenin (n = 25), or N-cadherin-Δ-cyto (n = 27). α3β4-myc nAChRs pixel area was measured within the entire cell-cell contact area and divided by the length of the contact. Pixel density in the cell contact area between cells expressing mEGFP was considered 100%. E) Ratio of α3β4-myc nAChRs pixel density (pixel area/µm membrane) between the cell-cell contact area and the contact-free cell membrane. C and D) CHO cells expressing N-cadherin and α3β4-myc nAChRs were treated with DMSO 10 µl/ml or with the indicated drug: LAT-A 10 µM, CTCH-D 2 µM, RhoA Inh 1 µg/ml, and Y27632 10 µM. C) Effect of pharmacological treatments on α3β4-myc nAChRs (pixel area/µm membrane) at N-cadherin-mediated cell-cell contacts as compared to cell-cell contacts between untreated cells expressing mEGFP and α3β4-myc nAChRs (100%). D) Ratio between α3β4-myc nAChRs (pixel area/µm membrane) within cell-cell contacts and contact-free cell membrane in N-cadherin-mediated cell-cell contacts treated with the indicated drug as compared to cell-cell contacts between cells expressing mEGFP (100%). Values represent the mean ± SEM of each experimental group (mEGFP, n = 24; DMSO, n = 20; LAT-A, n = 21; CTCH-D, n = 16; RhoA Inh, n = 21; and ROCK, n = 21). One-way ANOVA in A, B, C, and D p<0.001. Post-hoc Bonferroni test comparisons with mEGFP, *p<0.05; horizontal bars indicate comparisons with N-cadherin (A and B) and N-cadherin plus DMSO *p<0.05 (C and D).
(TIF)

Figure S2 Cell-cell contact length is not significantly affected by deletions of N-cadherin cytoplasmic domain and by inhibitors of actin polymerization, RhoA, and ROCK. A) Analysis of cell-cell contact length in cells expressing mEGFP (n = 24), N-cadherin (n = 22), N-cadherin-Δ-β-catenin (n = 25), or N-cadherin-Δ-cyto (n = 27). The length of the cell-cell contact was measure on the confocal images used for analyzing the cell surface distribution of nAChRs. B) Analysis of cell-cell contact length in cells expressing N-cadherin and treated with DMSO 10 µl/ml (n = 20), LAT-A 10 µM (n = 21), CTCH-D 2 µM (n = 15), RhoA Inh 1 µg/ml (n = 21), or Y27632 10 µM (n = 21). No statistical significant differences were found between groups. One-way ANOVA, p = 0.5.
(TIF)

Figure S3 Analysis of α3β4-myc nAChR expression levels on cells expressing mEGFP, N-cadherin, N-cadherin-Δ-β-cat (Δ-β-cat), and N-cadherin-Δ-cyto (Δ-cyto). Total gray value was measured on the contact free and contacting cell membranes of both cells and divided by the total membrane length (µm). No statistical significant differences were observed between groups indicating that α3β4-myc nAChR expression levels were not affected by the expression of N-cadherin deleted constructs. One-way ANOVA analysis, p>0.5.
(TIF)
