# Supplementary figures and images for: Cell Surface Localization of α3β4 Nicotinic Acetylcholine Receptors Is Regulated by N-Cadherin Homotypic Binding and Actomyosin Contractility
Source: PLoS One. 2013 Apr 23;8(4):e62435. doi: 10.1371/journal.pone.0062435 (PMC3633863; doi:10.1371/journal.pone.0062435)

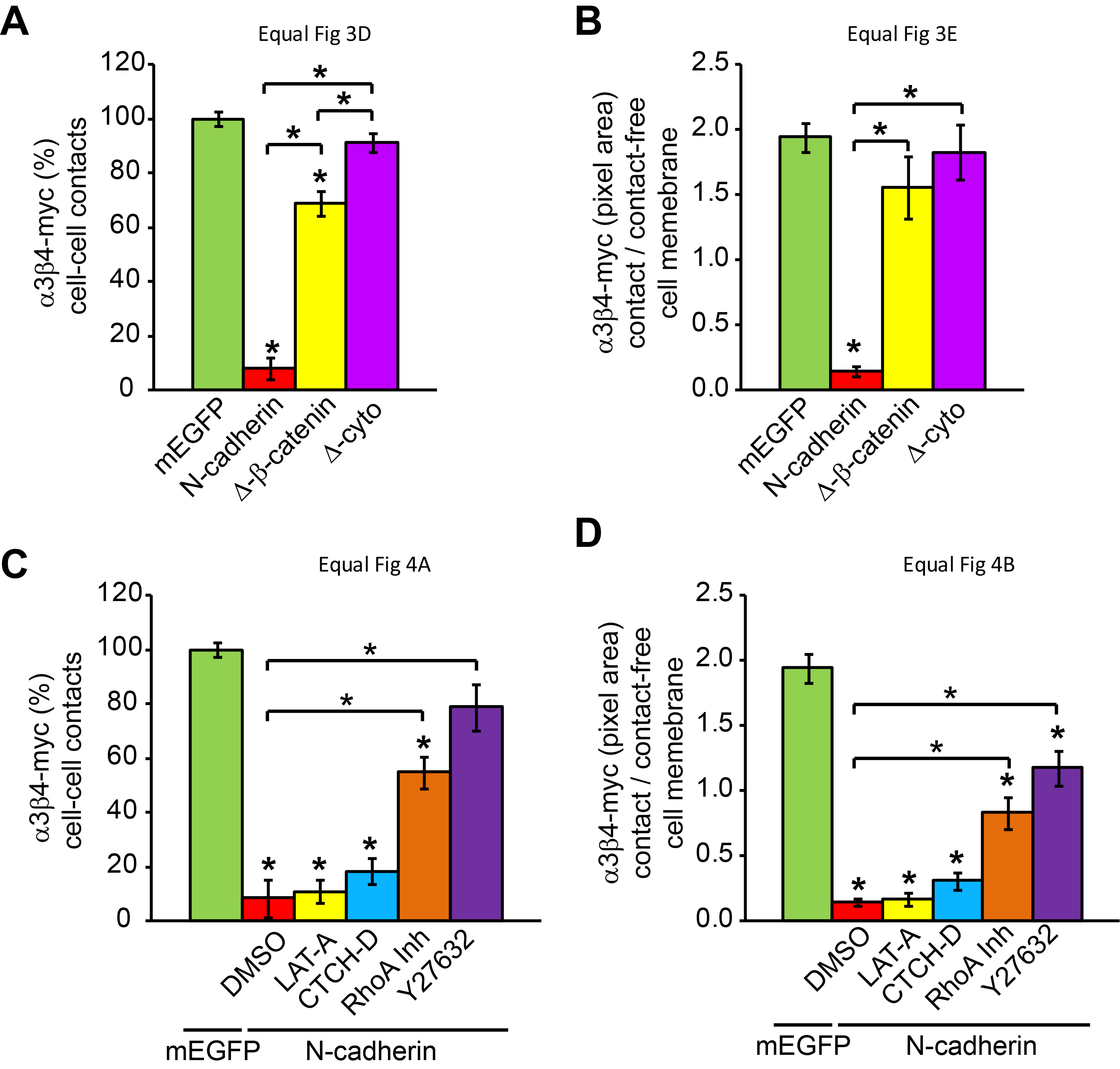

Supplement: Figure S1 — N-cadherin cytoplasmic domain is required to regulate α3β4-myc nAChRs localization on the cell surface. Pixel area was measured in the entire cell-cell contact membrane and the entire contact-free cell membrane and normalized to membrane length (µm). A) Analysis of pixel area of α3β4-myc nAChRs per µm of cell membrane within the cell-cell contact area in CHO cells expression mEGFP (n = 24), N-cadherin (n = 22), N-cadherin-Δ-β-catenin (n = 25), or N-cadherin-Δ-cyto (n = 27). α3β4-myc nAChRs pixel area was measured within the entire cell-cell contact area and divided by the length of the contact. Pixel density in the cell contact area between cells expressing mEGFP was considered 100%. E) Ratio of α3β4-myc nAChRs pixel density (pixel area/µm membrane) between the cell-cell contact area and the contact-free cell membrane. C and D) CHO cells expressing N-cadherin and α3β4-myc nAChRs were treated with DMSO 10 µl/ml or with the indicated drug: LAT-A 10 µM, CTCH-D 2 µM, RhoA Inh 1 µg/ml, and Y27632 10 µM. C) Effect of pharmacological treatments on α3β4-myc nAChRs (pixel area/µm membrane) at N-cadherin-mediated cell-cell contacts as compared to cell-cell contacts between untreated cells expressing mEGFP and α3β4-myc nAChRs (100%). D) Ratio between α3β4-myc nAChRs (pixel area/µm membrane) within cell-cell contacts and contact-free cell membrane in N-cadherin-mediated cell-cell contacts treated with the indicated drug as compared to cell-cell contacts between cells expressing mEGFP (100%). Values represent the mean ± SEM of each experimental group (mEGFP, n = 24; DMSO, n = 20; LAT-A, n = 21; CTCH-D, n = 16; RhoA Inh, n = 21; and ROCK, n = 21). One-way ANOVA in A, B, C, and D p<0.001. Post-hoc Bonferroni test comparisons with mEGFP, *p<0.05; horizontal bars indicate comparisons with N-cadherin (A and B) and N-cadherin plus DMSO *p<0.05 (C and D). (TIF) [file pone.0062435.s001.tif]

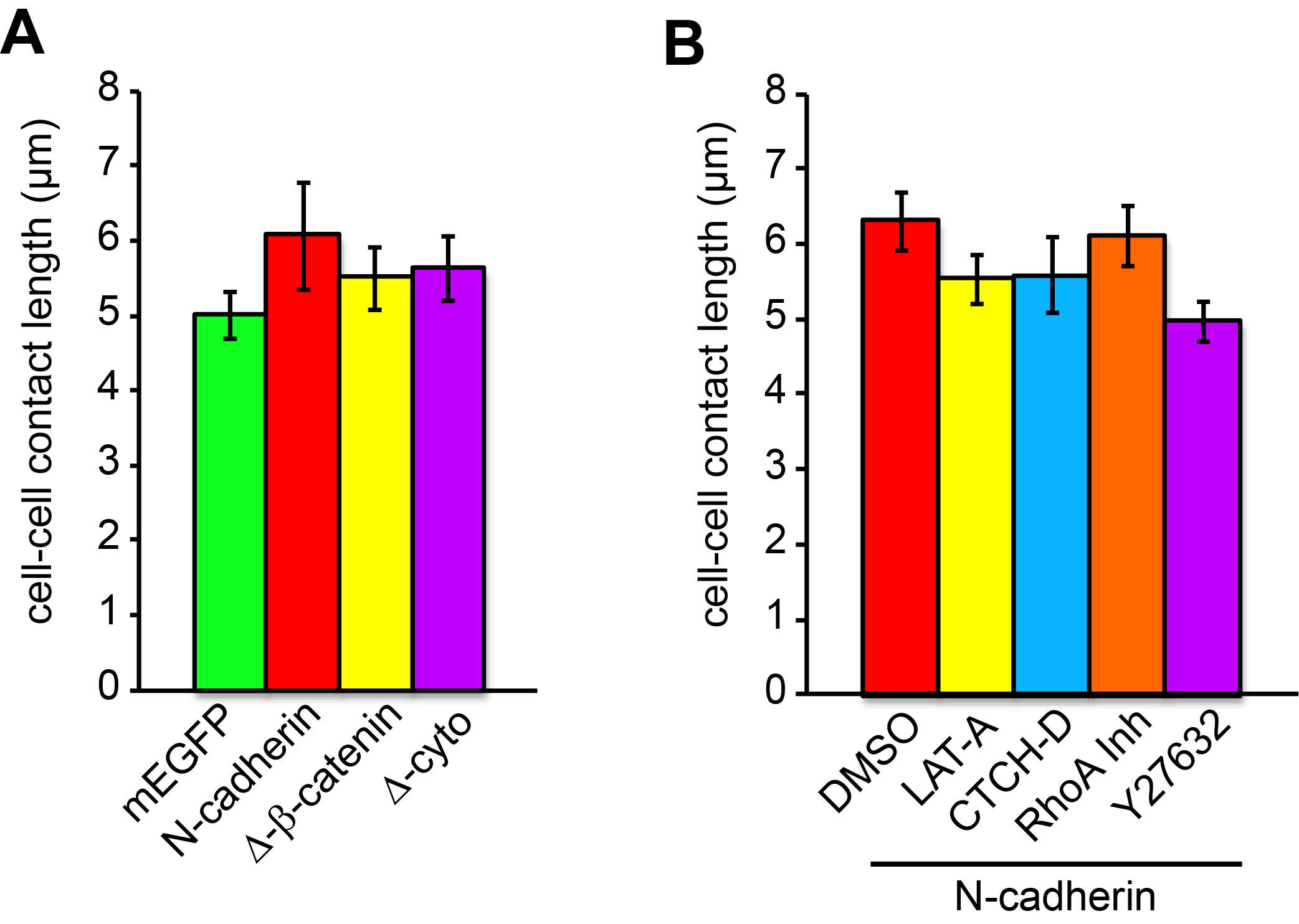

Supplement: Figure S2 — Cell-cell contact length is not significantly affected by deletions of N-cadherin cytoplasmic domain and by inhibitors of actin polymerization, RhoA, and ROCK. A) Analysis of cell-cell contact length in cells expressing mEGFP (n = 24), N-cadherin (n = 22), N-cadherin-Δ-β-catenin (n = 25), or N-cadherin-Δ-cyto (n = 27). The length of the cell-cell contact was measure on the confocal images used for analyzing the cell surface distribution of nAChRs. B) Analysis of cell-cell contact length in cells expressing N-cadherin and treated with DMSO 10 µl/ml (n = 20), LAT-A 10 µM (n = 21), CTCH-D 2 µM (n = 15), RhoA Inh 1 µg/ml (n = 21), or Y27632 10 µM (n = 21). No statistical significant differences were found between groups. One-way ANOVA, p = 0.5. (TIF) [file pone.0062435.s002.tif]

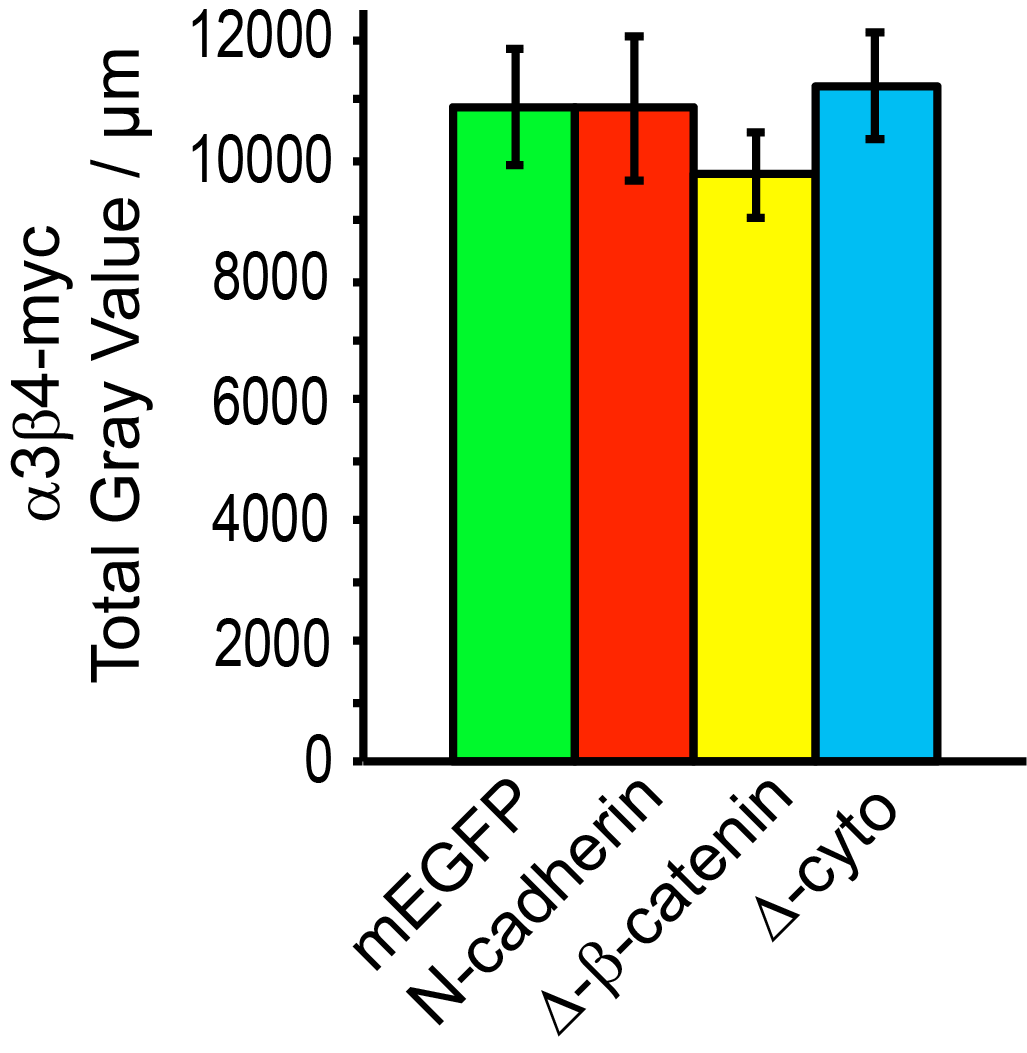

Supplement: Figure S3 — Analysis of α3β4-myc nAChR expression levels on cells expressing mEGFP, N-cadherin, N-cadherin-Δ-β-cat (Δ-β-cat), and N-cadherin-Δ-cyto (Δ-cyto). Total gray value was measured on the contact free and contacting cell membranes of both cells and divided by the total membrane length (µm). No statistical significant differences were observed between groups indicating that α3β4-myc nAChR expression levels were not affected by the expression of N-cadherin deleted constructs. One-way ANOVA analysis, p>0.5. (TIF) [file pone.0062435.s003.tif]
